# Development of a Cardiovascular Disease Risk Prediction Model: A Preliminary Retrospective Cohort Study of a Patient Sample in Saudi Arabia

**DOI:** 10.3390/jcm12155115

**Published:** 2023-08-04

**Authors:** Khaled Alabduljabbar, Mohammed Alkhalifah, Abdulaziz Aldheshe, Abdulelah Bin Shihah, Ahmed Abu-Zaid, Edward B. DeVol, Norah Albedah, Haifa Aldakhil, Balqees Alzayed, Ahmed Mahmoud, Abdullah Alkhenizan

**Affiliations:** 1 Department of Family Medicine & Polyclinics, King Faisal Specialist Hospital and Research Centre, Riyadh 11211, Saudi Arabia; alkhalifahmk@outlook.com (M.A.); aldheshe@gmail.com (A.A.); abdulelah_bin_shihah@hotmail.com (A.B.S.); mahmedsherif@kfshrc.edu.sa (A.M.); 2 College of Medicine, Alfaisal University, Riyadh 11533, Saudi Arabia; aabuzaid@live.com; 3 College of Graduate Health Sciences, University of Tennessee Health Science Center, Memphis, TN 38163, USA; 4 Department of Epidemiology and Scientific Computing, King Faisal Specialist Hospital and Research Centre, Riyadh 11211, Saudi Arabia; edevol@kfshrc.edu.sa (E.B.D.); nmalbedah@kfshrc.edu.sa (N.A.); hialdakhil@kfshrc.edu.sa (H.A.); balzayed@kfshrc.edu.sa (B.A.)

**Keywords:** retrospective, cardiovascular disease, risk prediction model, cardiovascular risk assessment, cardiac outcomes, primary health care, Saudi population, Saudi Arabia

## Abstract

Saudi Arabia has an alarmingly high incidence of cardiovascular disease (CVD) and its associated risk factors. To effectively assess CVD risk, it is essential to develop tailored models for diverse regions and ethnicities using local population variables. No CVD risk prediction model has been locally developed. This study aims to develop the first 10-year CVD risk prediction model for Saudi adults aged 18 to 75 years. The electronic health records of Saudi male and female patients aged 18 to 75 years, who were seen in primary care settings between 2002 and 2019, were reviewed retrospectively via the Integrated Clinical Information System (ICIS) database (from January 2002 to February 2019). The Cox regression model was used to identify the risk factors and develop the CVD risk prediction model. Overall, 451 patients were included in this study, with a mean follow-up of 12.05 years. Thirty-five (7.7%) patients developed a CVD event. The following risk factors were included: fasting blood sugar (FBS) and high-density lipoprotein cholesterol (HDL-c), heart failure, antihyperlipidemic therapy, antithrombotic therapy, and antihypertension therapy. The Bayesian information criterion (BIC) score was 314.4. This is the first prediction model developed in Saudi Arabia and the second in any Arab country after the Omani study. We assume that our CVD predication model will have the potential to be used widely after the validation study.

## 1. Introduction

Cardiovascular disease (CVD) is one of the most common non-communicable diseases and the main cause of death worldwide [1,2]. The burden of CVD is increasing in prevalence in developing countries [1]. In the Saudi population, the estimated prevalence of cardiovascular disease (CVD) is approximately 5.5% [3]. More than 50% of CVD mortality was estimated to be caused by the main modifiable risk factors, namely hypertension, diabetes mellitus, hyperlipidemia, obesity, and smoking [4]. In a World Health Organization report, it is estimated that about 37% of deaths from non-communicable diseases in all ages are caused by CVD in Saudi Arabia [5].

The prevalence of diabetes mellitus and hypertension among the Saudi population is 10.1% and 13.5%, respectively [6,7]. It is also estimated that the percentage of smokers has reached 14% of the total population (15 years and above) in Saudi Arabia [8]. In the United Arab Emirates, 28.4% of the population was found to have a Framingham risk score >20% in one cross-sectional community-based study [9].

Current recommendations on the prevention of CVD focus on the need to reduce the total cardiovascular risk of an individual rather than the presence of any particular risk factor [10,11]. For this reason, estimating the risk of cardiovascular events using statistical equations has drawn the interest of many researchers in the last few decades. Multiple risk prediction models and charts have been developed and utilized in clinical practice for the prevention, early detection, and management of CVD. Examples of prediction models include the atherosclerotic cardiovascular disease (ASCVD) risk calculator recommended by the American College of Cardiology/American Heart Association, Framingham risk assessment score, and QRISK assessment score that was updated in 2017 [11,12,13]. There is a significant need for prediction models that target Arab populations [14]. Recently, the first Arabic model was developed and validated, specifically for Omani individuals with type 2 diabetes mellitus, based on a retrospective cohort study with a sample size of 2039 patients [15,16].

Recent evidence has shown that using risk prediction models leads to better outcomes in risk management and prevention. Optimal CVD risk assessment for individuals within a specific population requires the development of different risk assessment models specific to different regions and ethnicities based on variables measured from these local populations. One model cannot accurately estimate CVD risk in different populations [17].

In view of the fact that Saudi Arabia has an alarmingly high incidence of CVD and its associated risk factors, and that, to the best of our knowledge, no CVD risk prediction model has been locally developed, it is vitally important that a specific risk assessment tool be created for the Saudi population. Such a model will help shape local CVD prevention and management strategies. To fulfill this need, this study was initiated, with the aim of developing the first 10-year CVD risk prediction model for Saudi adults aged 18 to 75 years.

## 2. Materials and Methods

### 2.1. Subject Identification and Data Abstraction

A retrospective chart review of the electronic health record from the Integrated Clinical Information System (ICIS) database of Saudi male and female patients aged 18 to 75 years who were seen in the Family Medicine & Polyclinics Department at King Faisal Specialist Hospital and Research Centre (KFSH&RC) in Riyadh between February 2002 and December 2019 was conducted. The study was approved by the Research Ethics Committee of KFSH&RC (RAC# 2191-071).

### 2.2. Sample Size

Our sample size estimation was based on a comparison between CVD in the diabetic and non-diabetic groups. According to the literature, the two-year event rate for CVD is 10% among non-diabetics and about 45% among diabetics [18]. The total number of subjects that we needed to recruit in the study to detect a hazard ratio (HR) of 3 (this is the null hypothesis) for a type I error rate of 5% and power of 80% was 350 subjects [19]. HR is a measure of how often a particular event happens in one group compared to how often it happens in another group over time.

### 2.3. Variables

The collected data included the following variables (listed in Appendix A): demographics such as age, gender, region of residence, marital status, smoking history, and employment status. Additionally, the average height, weight, and body mass index (BMI), as well as the average of 5 readings from different years of blood pressure, lipid profile, fasting glucose, hemoglobin A1C, and estimated glomerular filtration rate (eGFR), were collected.

During the chart review, any confirmed physician diagnosis of the following diseases at any point in the follow-ups was also recorded: hypertension, diabetes mellitus, dyslipidemia, heart failure, rheumatoid arthritis, atrial fibrillation, albuminuria, and chronic kidney disease (CKD). In addition, any history of premature (women less than 65 years and men less than 55 years) cardiovascular events in a first-degree relative, which includes parents, offspring, and siblings, was noted. Information about any medications used during the follow-up period, including antihypertensive, antihyperlipidemic, antidiabetic, or antithrombotic drugs, was collected.

The outcome would be defined as the first fatal or non-fatal CVD event confirmed and recorded by a physician, including the following: coronary heart disease (stable angina, unstable angina, or acute myocardial infarction) and stroke (ischemic or hemorrhagic). Any patient with a confirmed diagnosis of CVD, heart failure, or end-stage renal disease prior to 2002 was excluded.

### 2.4. Statistical Analysis

The statistical analyses of the data were carried out with a combination of the following tools: JMP version 14.0 (Cary, NC, USA) and Stata version 17.0 (College Station, TX, USA). Categorical variables were presented as proportions. Continuous variables were expressed as means and standard deviations (SDs). The level of statistical significance was set at *p* < 0.05. We fit Cox survival analyses to find the best model based on the Akaike information criterion (AIC) and the Bayesian information criterion (BIC), which assess and compare the performance and parsimony of competing models. Both the AIC and the BIC are information theory-based measures for model selection and are commonly used. Typically, the two measures agree with each other for model selection. They balance the two features of bias and variance. They differ quantitatively in terms of an added penalty for the BIC. Other model selection techniques (e.g., Lasso) were not considered with the assumption that they would not add significantly to the results [20,21]. Cox regression modeling was used to identify independent risk factors associated with CVD and to develop the CVD risk prediction model using the manual addition and deletion method. The missing data were handled using the complete case analysis (CCA) method.

### 2.5. Construction of the Model

The Cox regression model was used to identify the associated risk factors with CVD and to develop the CVD risk prediction model. Univariate analysis for all 32 variables was done to determine which risk factor would be included in the model; variables that tended to be significant were taken to create a multivariate model. More than 10 multivariate models were created. To select the best-fitting model, the Bayesian Information Criterion (BIC) was used in which a lower BIC value indicates a better model. The final model included 6 independent risk factors (i.e., FBS, HDL-c, heart failure, antihyperlipidemic therapy, antithrombotic therapy, and antihypertension therapy), and the BIC was 314.4.

### 2.6. Scoring System

In this study, longitudinal data were gathered from 451 patients from a family medicine outpatient service. To facilitate the use of the prediction model in daily practice, a point system was formulated. This system is based on the methods of Sullivan et al. [22]. The categorization of the continuous variable was guided by clinical significance, with the reference value determined as the mid-point for each category. The remaining risk factors were modeled using sets of indicator variables (0,1). The referent risk factor profile was chosen to be an individual with FBS of 5.6 and total HDL-c of 2, without a history of heart failure, no antihyperlipidemic therapy, no antihypertensive, and no antithrombotic. The inter-category distances were determined in terms of regression units for each risk factor. A constant was applied to each inter-category distance in order to derive a point and determine the risk estimate (probability of developing an event over the predetermined time frame) based on the total points across the risk factors. This constant will reflect an increase in the risk associated with one unit increase in the FBS point. The derived point will be rounded to a whole number. The theoretical range of this point system will range from 0 to 47.

## 3. Results

Between 2002 and 2012, a total of 451 Saudi male and female patients who were seen in the Family Medicine & Polyclinics Department at King Faisal Specialist Hospital and Research Centre (KFSH&RC) in Riyadh were reviewed retrospectively. Table 1 displays the distribution of risk factor characteristics among the sample at baseline. The mean age was 43.9 years, and 35 patients developed CVD events during the study period. The mean FBS at baseline was 6.15 mmol/L. The majority of the studied patients were non-smokers and had no family history of premature CVD.

Table 2 presents the frequency and percentage of the lipid panel results. Over a quarter (26.14%) of the patients had borderline LDL-c levels, while the majority (58.41%) had normal HDL-c levels. Nearly three quarters (72.27%) had normal triglyceride levels.

**Figure 1:**
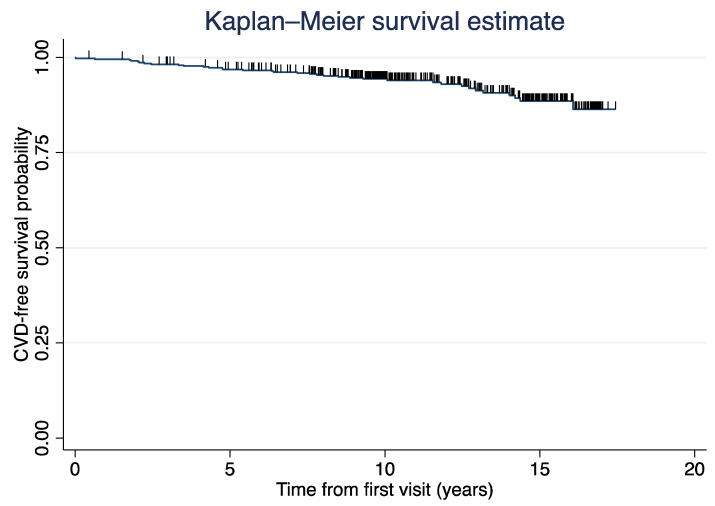
Kaplan-Meier curve of cardiovascular disease (CVD) events.

Based on a univariate analysis of 32 clinically relevant variables (Table S1), six variables were found to be significantly associated with CVD events (*p* < 0.05) and included in the best-fitting multivariate model presented in Table 3. The predictors of CVD were FBS, HDL-c, heart failure, antihyperlipidemic therapy, antithrombotic therapy, and antihypertensive therapy. The table presents the coefficients (also known as betas) of the Cox proportional hazards model, along with the means (or proportions positive for each risk-factor category). In Table 3, the beta value represents the estimated regression coefficient for each predictor of CVD. A positive beta value indicates that an increase in the predictor variable is associated with an increased risk of developing CVD, and vice versa. Heart failure, antihyperlipidemic therapy, antithrombotic therapy, and antihypertensive therapy were analyzed as time-varying covariates, and the proportions considered that the occurrence of the covariate (e.g., heart failure) might happen after the cardiovascular event and therefore should not be counted. The average 10-year event-free rate was 94.5%. During the 10-year follow-up, 35 (7.7%) of the 451 participants developed cardiovascular events (as shown in Figure 1).

Table 4 presents the points assigned to the variables used to estimate the multivariate risk of CVD. An illustration of using the point system is provided in Appendix B, and the risk estimation with corresponding points is shown in Table 5.

## 4. Discussion

This is the first CVD risk prediction tool in Saudi Arabia. No previous CVD risk prediction tool has been developed specifically for the Saudi Arabian population. The cumulative incidence was 7.7% in this study. Accurate assessment of cardiovascular risk is essential to effectively weigh the risks and benefits of treatment. The American College of Cardiology’s ASCVD risk assessment tool and the Framingham calculator are well-trusted and validated tools universally, but they are more accurate when used for the population they were developed for. Both tools have been found to significantly overestimate cardiovascular risk in multi-ethnic cohorts of patients [23]. The Korean heart study included 200,000 Korean adults [24]. They also found that the American College of Cardiology’s ASCVD equations overestimated ASCVD risk in Korea, and that the Korean risk prediction model showed the best predictive capability for cardiovascular risk. The ACC calculator was derived from patient cohorts in the 1970s and 1980s, which may be another reason for overestimation in this cohort [25]. Therefore, we developed the fundamental cornerstone of a CVD risk prediction tool for the Saudi Arabian population. Herein, it has been developed to pave the way for similar studies.

The Framingham heart study was initially conducted on 5209 patients over a 6-year interval. The included risk factors comprised age, gender, blood pressure, LDL, and HDL cholesterol, smoking, glucose status, and cardiac enlargement [26]. The ASCVD risk calculator included 13 predictors; furthermore, the newest version of the UK Prospective Diabetes Study (UKPDS) included 13 predictors [11,27]. In contrast, the variables in our study included FBS, HDL-c, use of antihypertensive therapy, antihyperlipidemic therapy, antithrombotic therapy, and heart failure. Since then, many other risk models have been developed. They differ in various aspects, including the types of populations, endpoints, and predictor variables, leading to widely varying risk estimates [25]. In our study, we incorporated stroke into the CVD outcome.

In consonance with the ASCVD risk calculator [11], we found that diabetes mellitus and low HDL-c levels are significantly associated with CVD. We found that participants on antihypertensive therapy were at higher risk of developing an event compared with those who were not. In contrast, UKPDS revealed that being on an antihypertensive medication decreases the risk of developing cardiovascular events in the general population.

Compared with the ASCVD risk calculator or Qrisk3 tool, which is currently used in Saudi Arabia, our tool assigns 11 points for FBS > 7.0 mmol/L and 13 points for HDL-c level < 1.03 mmol/L in our point system.

Additionally, to facilitate the use of this tool by clinicians, it can be converted into a program or an application. Further studies are needed to validate its accuracy and applicability among the Saudi Arabian population.

One limitation of our study is that it was conducted on a relatively small, restrictive, and narrow sample size and did not include all regions of Saudi Arabia, which may limit the generalizability of the results. Besides, our sample size estimation was based on a comparison between CVD in diabetic and non-diabetic groups, and the estimation of sample size could have been impacted by the choice of other risk factors, such as the presence and absence of hypertension or dyslipidemia. Additionally, the number of events was small, which could have likely impacted the true effect size and the power of analysis. The lack of external validation of our model to gauge its potential transferability to other cohorts of Saudi patients is a noteworthy limitation that should be acknowledged. An additional shortcoming is that our developed model was not compared to validated and generally accepted international risk score applications from Europe and America. Besides, out of the 6 independent risk factor variables that were included in the final prediction model, the use of the development of heart failure during the follow-up period as an explanatory variable suggests that this analysis is a time-dependent Cox proportional hazards model, and it would have been better to be analyzed as such. Lastly, this study identified antihypertension therapy as a CVD risk factor. However, the usefulness of using antihypertension therapy as a CVD risk factor prediction may be questionable, as antihypertensive use is so heterogeneous. For example, some use only 1 drug, some use more than 3 drugs, and some antihypertensive users have their blood pressure under control, whereas others do not.

However, the present study does add a valuable prediction model, as the sample size calculation was representative. The Omani and Australian tools were exclusively used for type 2 diabetes mellitus patients, and they had larger sample sizes than ours [15,28]. However, their follow-up period was 5 years, which is half of ours. Another limitation is the lack of documentation of the lifestyle history, such as diet and physical activity, that we cannot rule out its contribution in CVD. However, it is worth noting that many other international risk prediction tools also do not include lifestyle history in their models.

Clearly, the accuracy of risk estimation models will be negatively affected if the models are applied to populations different from the one they were derived from, or to the same population, but at a later time when significant changes in cardiovascular mortality may have occurred. In such situations, it becomes critically important to derive a new model from recent local cohorts of patient [29]. Cardiovascular risk assessment depends on risk factor profile, as well as average CVD risk in the specified population, and risk-factor levels in the population [17].

Further prospective cohort studies need to be developed in the future to better model our local population, with particular care to include a large population of older age and higher event rates. Following this, external validation is an essential step to ensure the transferability of the model—i.e., that it can be applied to other cohorts of patients, and not only the derivation cohort. The conclusion that may be drawn is that clinicians should think twice before applying commonly used CVD risk prediction equations for ASCVD risk stratification in specific populations.

## 5. Conclusions

As of today, no CVD risk prediction model has been locally developed in Saudi Arabia. Thus, in this research, we endeavored to develop the first 10-year CVD risk prediction model for 451 Saudi adults aged 18 to 75 years who attended the Family Medicine & Polyclinics Department at King Faisal Specialist Hospital and Research Centre (KFSH&RC) in Riyadh. Methodologically, the Cox regression model was used to identify the risk factors and establish the CVD risk prediction model. Key limitations of the CVD risk prediction presented model comprise the preliminary nature of the report, the small sample size, and all patients recruited from a single institute. Prospective research includes conducting large prospective cohort studies to better model our local population, followed by external validation studies to guarantee national transferability of the model to the larger population of Saudi Arabia. All in all, we believe that our CVD predication model has significant potential to be widely used in clinical practice after undergoing the validation study.

## Figures and Tables

**Table 1:** Descriptive statistics of risk factor characteristics of the studied sample (*n* = 451).

Characteristic	Frequency/Mean	%
Age (years), mean ± SD	43.9 ± 15.5	
BMI (kg/m^2^), mean ± SD ^1^	30.8 ± 6.5	
FBS (mmol/L) ^2^ <5.6 5.6 < FBS < 7.0 >7.0	224 129 71	52.83 30.42 16.75
Gender Male Female	181 270	40.13 59.87
Smoking status ^3^ Current smoker Former smoker Never smoked	44 6 290	12.94 1.76 85.29
Family history of premature CVD	6	1.33
CVD events	35	7.7
Chronic kidney disease	25	5.54
Rheumatoid arthritis	3	0.67
Antidiabetic therapy	172	38.14
Antihyperlipidemic therapy	199	55.88
Antithrombotic therapy	131	29.05
Antihypertensive therapy	207	45.90
Heart failure	10	2.22

^1^ 77 missing answers, ^2^ 27 missing answers, ^3^ 11 missing answers. n: number; SD: standard deviation; FBS: fasting blood sugar; BMI: body mass index; CVD: cardiovascular disease.

**Table 2:** Lipid panel frequency and percentage of the studied sample (*n* = 451).

Lipid Profile Range	Frequency	%
LDL-c ^1^ <2.59 mmol/L 2.59–3.36 mmol/L 3.37–4.14 mmol/L 4.15–4.90 mmol/L ≥4.90 mmol/L	116 172 115 31 6	26.36 39.09 26.14 7.05 1.36
HDL-c ^1^ <1.04 mmol/L 1.04–1.55 mmol/L >1.55 mmol/L	74 257 109	16.82 58.41 24.77
Triglycerides ^1^ <1.7 mmol/L 1.7–2.25 mmol/LL 2.26–5.64 mmol/L ≥5.65 mmol/L	318 66 54 2	72.27 15 12.27 0.45
Total cholesterol ^1^ <5.2 mmol/L 5.2–6.2 mmol/L ≥6.2 mmol/L	281 129 30	63.86 29.32 6.82

^1^ 11 missing answers. n: number; LDL-c: low-density lipoprotein cholesterol; HDL-c: high-density lipoprotein cholesterol.

**Table 3:** Parameters (*β* s) of the multivariate Cox regression model.

Risk Factor	HR (95% CI)	β	Mean	SE	*p*-Value
FBS (mmol/L)	1.21 (1.11–1.32)	0.199	6.379	0.042	0.000
HDL-c (mmol/L)	0.13 (0.03–0.48)	−1.98	1.331	0.643	0.002
Heart failure	3.59 (1.20–10.74)	1.35	0.015	0.555	0.015
Antihyperlipidemic therapy	3.17 (1.27–7.93)	1.14	0.312	0.465	0.014
Antithrombotic therapy	2.34 (1.02–5.37)	0.79	0.206	0.425	0.062
Antihypertension therapy	3.20 (1.14–8.99)	1.22	0.375	0.528	0.021

HR: hazard ratio; CI: confidence interval; β: regression coefficient; SE: standard error; FBS: fasting blood sugar; HDL-c: high-density lipoprotein cholesterol.

**Table 4:** Cardiovascular disease risk estimation points.

Points Assigned	FBS (mmol/L)	HDL-c (mmol/L)	Antihyperlipidemic Therapy	Antithrombotic Therapy	Antihypertension Therapy	Heart Failure
0	<5.6	>1.55	No	No	No	No
1						
2	5.6 < FBS < 7.0					
3						
4				Yes		
5						
6			Yes		Yes	
7		1.03 < HDL-c < 1.55				Yes
8						
9						
10						
11	>7.0					
12						
13		<1.03				

FBS: fasting blood sugar; HDL-c: high-density lipoprotein cholesterol.

**Table 5:** The risk estimation of cardiovascular disease with the corresponding points.

Points	Risk Estimate %	Points	Risk Estimate %
0	0.344996151	29	67.00661523
1	0.420798054	30	74.15401108
2	0.513212075	31	80.81275049
3	0.625857836	32	86.66033095
4	0.763133414	33	91.43913254
5	0.930377765	34	95.01715431
6	1.134064574	35	97.42554408
7	1.382032388	36	98.84986966
8	1.683755981	37	99.56971269
9	2.050663558	38	99.87035528
10	2.496503385	39	99.97000592
11	3.037761245	40	99.99497261
12	3.6941262	41	99.99943134
13	4.48899561	42	99.99996019
14	5.45	43	99.99999845
15	6.609512655	44	99.99999997
16	8.00508574	45	100
17	9.679722051	46	100
18	11.68184697	47	100
19	14.06478727		
20	16.88549319		
21	20.20216307		
22	24.07036169		
23	28.53719432		
24	33.63316234		
25	39.36156659		
26	45.68584913		
27	52.51618369		
28	59.69798186		

## Data Availability

Data are available upon request.

## Supplementary Materials

The following supporting information can be downloaded at: https://www.mdpi.com/article/10.3390/jcm12155115/s1, Table S1: Fit indices for multivariate Models.

## Appendix A

The potential risk factors that were collected.

The potential risk factors that were collected:
1. Age (years). 2. Gender: 1—Male, 2—Female. 3. Region of residence: 1—Riyadh, 2—Makkah, 3—Eastern Province, 4—AlMadinah, 5—AlBahah, 6—Northern Borders, 7—AlJawf, 8—AlQasim, 9—Asir, 10—Hail, 11—Jizan, 12—Najran, 13—Tabuk. 4. Marital status: 1—Single, 2—Married, 3—Divorced, 4—Widow. 5. Employment Status: 1—Employed, 2—Self-employed, 3—Unemployed, 4—Military, 5—Retired. 6. Height (cm). 7. Weight (kg). 8. Body Mass Index (kg/m^2^). 9. Systolic blood pressure (mmHg). 10. Diastolic blood pressure (mmHg). 11. Smoking status: 1—Current smoker, 2—Former smoker, 3—Never smoked 4—Unknown. 12. First-degree family history of premature cardiovascular disease: 0—No, 1—Yes.
Any history of the following diseases. If yes, specify the date of diagnosis:
13. Type 1 diabetes: 0—No, 1—Yes. 14. Type 2 diabetes: 0—No, 1—Yes. 15. Hypertension: 0—No, 1—Yes. 16. Heart failure: 0—No, 1—Yes. 17. Hyperlipidemia: 0—No, 1—Yes. 18. Chronic kidney disease: 0—No, 1—Yes. 19. Rheumatoid arthritis: 0—No, 1—Yes. 20. Atrial fibrillation: 0—No, 1—Yes. 21. Albuminuria: 0—No, 1—Yes.
Medications information:
22. Current antihypertensive medication? 0—No, 1—Yes 23. Current antihyperlipidemic medication? 0—No, 1—Yes 24. Current antidiabetic medication? 0—No, 1—Yes 25. Current antithrombotic medication? 0—No, 1—Yes
Lab results:
26. Fasting blood glucose (mmol/L). 27. HbA1C (%) 28. Triglyceride (mmol/L). 29. Low-density lipoprotein cholesterol (mmol/L). 30. High-density lipoprotein cholesterol (mmol/L). 31. Total cholesterol (mmol/L). 32. Estimated glomerular filtration rate (eGFR) (mL/min/1.73m^2^).

## Appendix B

An illustration of using the point system.

Case:

Patient X with FBS of 6.8 and total HDL-c of 1.4, taking antihypertensive and antithrombotic therapy, no anti-hyperlipidemic therapy, and with no history of heart failure.

Risk factor	Value	Points
FBS	6.8	2
HDL-c	1.4	7
Anti-hypertensivetherapy	Yes	6
Antithrombotic therapy Antihyperlipidemic therapy	Yes No	4 0
Heart failure	No	0
	Point total	19
	Estimate of risk	0.140647873

FBS: fasting blood sugar; HDL-c: high-density lipoprotein cholesterol.

The risk estimate based on the Cox model is computed as follows:

$$\sum_{i=1}^{p}\;\beta_{i}X_{i}|=0.199(6.8)+-1.985(1.4)+1.357(0)+1.14(0)+0.79(1)+1.22(1)=0.5912$$

$$\sum_{i=1}^{p}\;\beta_{i}{\bar{X}}_{i}=0.199\left(6.379\right)\pm 1.985\left(1.331\right)+1.357\left(0.015\right)+1.14\left(0.312\right)\;+0.79\left(0.206\right)+1.22\left(0.375\right)=-0.402$$

$$\hat{p}=1-S_{0}(t{)}^{\exp\left(\sum_{i=1}^{p}\;\beta_{i}X_{i}-\sum_{i=1}^{p}\;\beta_{i}{\bar{X}}_{i}\right)}=1-0.9455exp\;(0.5912-0.402)=0.140$$

The point system estimates a 10-year risk of 14.06%, whereas employing the Cox model directly gives 14.04%.
